# The cholesterol-lowering effects of oat varieties based on their difference in the composition of proteins and lipids

**DOI:** 10.1186/1476-511X-13-182

**Published:** 2014-12-05

**Authors:** Lina Guo, Li-Tao Tong, Liya Liu, Kui Zhong, Ju Qiu, Sumei Zhou

**Affiliations:** Institute of Agro-Products Processing Science and Technology, Chinese Academy of Agricultural Sciences/Key Laboratory of Agro-Products Processing, Ministry of Agriculture, 100193 Beijing, China; Institute of Food and Nutrition Development, Ministry of Agriculture, 12 Zhongguancun South Street, Haidian District, 100081 Beijing, China

**Keywords:** Oat hypocholesterolemic effect proteins lipids

## Abstract

**Background:**

The aim of present study is to investigate the hypocholesterolemic effects of the oat components other than the β-glucan in rats fed with a hypercholesterolemic diet.

**Methods:**

Four-week-old male Wister rats were divided into 6 groups of 7 rats each with similar mean body weights and serum cholesterol concentrations. Rats were fed with the experimental diets containing 10% oats flour for 30 days. Food intake was recorded and monitored everyday to ensure the similar contents of protein, starch, lipid and cellulose in all groups. The lipids levels in serum, liver, and faeces were determined.

**Results:**

The plasma total cholesterol concentrations in different oat groups were significantly reduced compared with the control group, and the effects were different among oat groups. The decrease extent of plasma total cholesterol and low-density lipoprotein-cholesterol concentrations increased with the increase of the proteins and lipids contents. Moreover, liver total cholesterol and cholesterol ester contents were markedly decreased. The fecal bile acids concentrations in the oat groups were significantly increased. Oat proteins had lower Lysine/Arginin (0.59 ~ 0.66) and Methionin/Glycine (0.27 ~ 0.35) ratio than casein (Lysine/Arginin, 2.33; Methionin/Glycine, 1.51). Oat lipids contained higher contents of total Vitamin E and plant sterols than that in soybean oil.

**Conclusion:**

These results indicated that dietary oat improved hypercholesterolemia by increasing the excretions of fecal bile acids, and this improvement was not only related to β-glucan, but also attributed to the lipids and proteins. Oat proteins decreased serum total cholesterol and low-density lipoprotein-cholesterol contents due to their low Lysine/Arginin and Methionin/Glycine ratio. The co-existence of oleic acid, linoleic, vitamin E, or plant sterols accounted for the hypocholesterolemic properties of oat lipids.

## Introduction

It is well known that dietary oat have been reported to reduce serum cholesterol [1] and obesity [2], prevent coronary heart disease [3], and improve symptoms of diabetes [4]. Numerous studies indicate that oat have high contents of β-glucan which is beneficial to human health, as it is considered to be responsible for these health benefits [5–7]. Oat contains 2.0 ~ 7.5% β-glucan [8], 13 ~ 20% protein [9], 2 ~ 12% crude fat [10], and about 60% starch [11]. Most of the studies focus on the health benefits of β-glucan, while the effects of other main components on reducing serum cholesterol are still unknown.

Numerous studies indicate that dietary plant proteins, such as soybean [12, 13], rice [14, 15] and buckwheat [16], reduce effectively serum cholesterol levels and prevent cardiovascular diseases. Compared with other cereals, oat contains higher content of protein, and the composition of oat amino acid is more reasonable [7]. Moreover, the levels of crude fat, showing the nutritional and functional potential [17], in oat are much higher than that of other cereal grain, which leads oat to become an excellent source of functional food [18]. Oat lipids are rich in polyunsaturated fatty acids, Vitamin E [7] and plan sterols [19]. Judd and Truswell concluded that both the lipophilic and lipophobic components of oat played a major role in decreasing serum cholesterol in humans [20]. However, there is no information about the effect of these components other than β-glucan in oat on the reduction of serum cholesterol in animals or humans.

In the present study, we determined the nutrient contents of different oat varieties, and selected five varieties. The five oat varieties have different contents of proteins and lipids, but the contents of β-glucan are similar, to investigate the cholesterol-lowing effects of the components other than β-glucan in Wistar-Lewis rats fed a hypercholesterolemic diet.

## Materials and methods

### Materials

Twenty-eight oat varieties, including huazao-No.2(hebei), Jinyan-No.8(shanxi), Jinyan-No.9(shanxi), pinyin-No.1(shanxi), baiyan-No.2(shanxi), bayou-No.8(shanxi), jinyan-No.13(youyu), bayou-No.8(youyu), yanke-No.1(neimenggu), caoyou-No.1(neimenggu), neiyan-No5(neimenggu), dingyou-No.1(gansu), dingyou-No.2(gansu), dingyou-No.3(gansu), dingyou-No.4(gansu), dingyou-No.5(gansu), dingyou-No.6(gansu), dingyou-No.7(gansu), dingyou-No.8(gansu), baiyan-No.4(jilin), baiyan-No.5(jilin), baiyan-No.8(jilin), bayan-No.2(sichuan), bayan-No.2(xinjiang), huawan-No.6(xinjiang), ningyou-No.1(ningxia), yanke-No1(ningxia), and baiyan-No.2(ningxia), were collected from main regions of oat cultivation in China between October and November, 2011. Oats were crushed into flour and then stored at 4°C for further study.

### Components determination

The contents of oat protein, lipid, β-glucan, ash and starch were determined based on GB/T5511-2008, GB/T14772-2008, AOAC995.16, GB/T 22510–2008 and AOAC 996.11, respectively. Amino acid compositions of oat proteins and casein were determined using an amino acid autoanalyzer (L-8900-type amino acid analyzer, Hitachi, Ltd., Japan) based on GB/T5009124-2003. Fatty acid compositions of oat lipids and soybean oil were determined by gas–liquid chromatography based on ISO 5508–1990. The Vitamin E content of oil was determined by high-performance liquid chromatography (HPLC) equipped with ultraviolet detector according to NY/T 1598–2008, C18 5 μm, 4.6 × 250 mm column was used. The sterol content of oil was determined by the method of UNI EN ISO 12228 (1999). The sterols were derivatized to trimethylsilyl ethers and its quantification by gas–liquid chromatography with a CP-Sil 5 CB capillary column was performed as follow: the column temperature was from 100°C to 235°C (10°C/min); 0.5 min isotherm; the column temperature was from 235°C to 300°C (10°C/min); 3 min isotherm; injector 300°C; flame ionization detector 320°C. Results mentioned above were on dry weight basis.

### Animals and diets

Four-week-old male Wister rats were purchased from Vital River Lab Animal Technology Co, Ltd. (Beijing, China). All animals were housed in cages in an air-conditioned room (temperature, 21-23°C; humidity, 55-65%; lights on, 08:00–20:00 h). After 7 days of acclimation, rats were divided into 6 groups of 7 rats each with similar mean body weights and serum cholesterol concentrations. Rats were fed with the experimental diets for 30 days. The compositions of experimental diets were prepared according to AIN-93G (American Institute of Nutrition 1993) with some modifications (Table 1). Oats (10% of diet) were added in the experimental diet. Food intake was recorded and monitored everyday to ensure the similar contents of protein, starch, lipid and cellulose in all groups. Body weights were recorded every three days. The rats were fasted for 16 h and then sacrificed by the removal of the whole blood from the abdominal aorta.

**Table 1:** **Composition of the experimental diets (g/1000 g diet)**

	Control	Jinyan-No.13	Bayou-No.8	Baiyan-No.2	Dingyou-No.8	Baiyan-No.8
Casein	200	182.70	181.06	180.6	180.6	180.0
Corn starch	397	328.9	330.8	331.6	332.2	333.3
Soybean oil	70	64.2	63.9	63.8	62.9	62.0
Cellulose	50	43.1	43.0	43.2	43.2	43.6
Sucrose	38.5	38.5	38.5	38.5	38.5	38.5
L-Cystine	3	3	3	3	3	3
Maltodextrin 10	132	132	132	132	132	132
t-Butylhydroquinone	0.014	0.014	0.014	0.014	0.014	0.014
Mineral mix	35	35	35	35	35	35
Vitamin mix	10	10	10	10	10	10
Choline bitartrate	2.5	2.5	2.5	2.5	2.5	2.5
Cholesterol	10	10	10	10	10	10
Lard	50	50	50	50	50	50
Cholate	2	2	2	2	2	2
Oat	0	100	100	100	100	100
Total	1000	1000	1000	1000	1000	1000

Diets were prepared based on AIN-93G recommendations.

All experiments were carried out according to the P.R. China legislation regarding the use and care of laboratory animals and were approved by the Bioethics Committee of the Institute of Medicinal Plant Development, Chinese Academy of Medical Sciences and Peking Union Medical College.

### Analysis of metabolic parameters in the rats

The contents of total cholesterol (TC), high-density lipoprotein-cholesterol (HDL-C), low-density lipoprotein-cholesterol (LDL-C), and triglyceride (TG) in serum were measured by Automatic Chemistry Analyzer (Hitachi, Tokyo, Japan). TC, TG and free cholesterol (FC) contents in liver were determined using enzymatic assay kits, including Tissue total cholesterol assay kit E1015, Tissue free cholesterol assay kit E1016 and Tissue triglyceride assay kit E1003-2, respectively, from Applygen Technologies Inc. (Beijing, China). Fecal bile acid content was measured according to the rats bile acids ELISA kit from Sen Shellfish Gamma Biotechnology Limited Company (NanJing Nanjing, China), and fecal cholesterol was measured using Tissue total cholesterol assay kit E1015. The fecal total lipid was determined by Soxhlet method.

### Statistical analysis

Data were expressed as means ± standard error of mean (*n* = 7) in rat study. Statistical analysis was performed by one-factorial analysis of variance (ANOVA) test. Significant differences (*P* < 0.05) were analyzed by Tukey–Kramer’s t test for *post-hoc* multiple comparisons.

## Results

**Figure 1:**
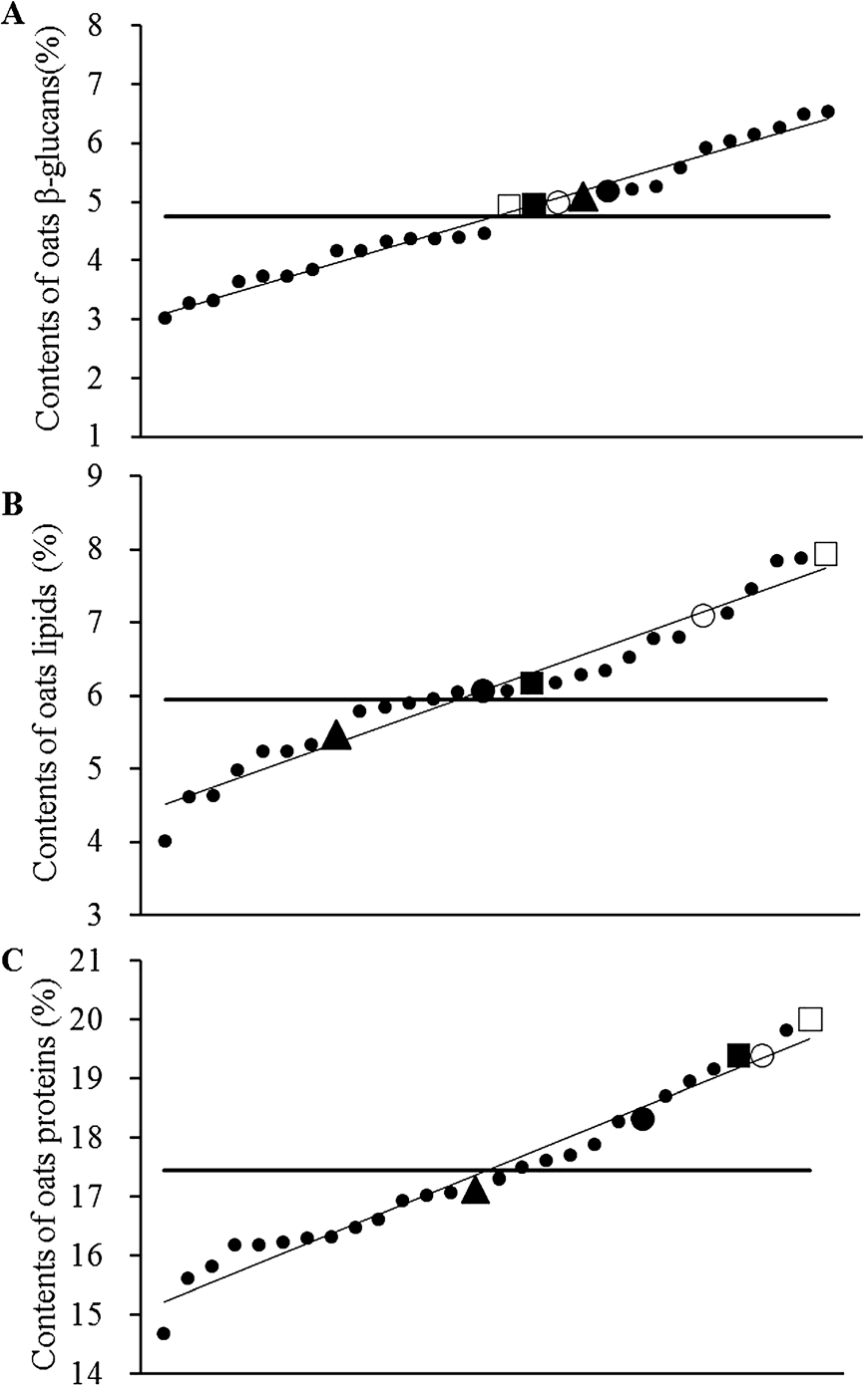
**The contents of β-glucan (A), lipids (B) and proteins (C) in twenty-eight oat varieties.** -represent the mean of β-glucan, lipid and protein contents.  Baiyan-No.8,  Dingyou-No.8,  Baiyan-No.2,  Bayou-No.8,  Jinyan-No.13,  other twenty-three oat varieties.

Figure 1 showed β-glucan, lipid and protein contents of twenty-eight oat varieties. The content of β-glucan ranged from 3.01% ~ 6.54%, and its average was 4.76%; the content of protein ranged from 14.66% ~ 19.99%, and its average was 17.44%; the content of lipid ranged from 4.02% ~ 7.94%, and its average was 5.95%. Five oat varieties, Jinyan-No.13, Bayou-No.8, Baiyan-No.2, Dingyou-No.8 and Baiyan-No.8, were selected based on Figure 1, which contained similar β-glucan content and gradient content of protein and lipid. The compositions of five oat varieties were shown in Table 2. The amino acid compositions of casein and oat proteins were shown in Table 3. As shown in Table 3, the ratio of lysine/arginin in oat proteins ranged from 0.59 ~ 0.66 and the ratio of methionin/glycine ranged from 0.27 ~ 0.35, both of which were lower in oat groups than those in control group. Table 4 showed the fatty acid compositions of the soybean oil and oats lipids, and indicated that the concentration of unsaturated fatty acids was higher in control group than that in oat groups. The tocopherol contents in different groups of oat and soybean oil were shown in Table 5. With the contents of total Vitamin E among five oat groups were almost the same, the total vitamin E contents in oat groups were higher than that in soybean oil. Table 6 showed the major plant sterol components of oats and soybean oil, which indicated the concentrations of total sterols in oats were basically consistent, but higher than that in soybean oil.

**Table 2:** **Analysis of oats composition (%)**

Groups	β-glucan	Proteins	Lipids	Starch	Ash
Jinyan-No.13	5.10 ± 0.11	17.30 ± 0.11	5.79 ± 0.11	58.37 ± 0.12	1.94 ± 0.05
Bayou-No.8	5.15 ± 0.07	18.94 ± 0.20	6.07 ± 0.36	56.86 ± 0.09	1.89 ± 0.09
Baiyan-No.2	4.99 ± 0.09	19.37 ± 0.03	6.17 ± 0.09	63.12 ± 0.13	2.29 ± 0.07
Dingyou-No.8	5.05 ± 0.12	19.39 ± 0.14	7.10 ± 0.67	53.01 ± 0.11	1.92 ± 0.09
Baiyan-No.8	4.92 ± 0.10	19.99 ± 0.04	7.94 ± 0.56	55.31 ± 0.17	1.90 ± 0.12

Data are means ± SD (n = 3).

**Table 3:** **Amino acid composition of dietary proteins (mg/g proteins)**

Amino acid	Casein	Jinyan-No.13	Bayou-No.8	Baiyan-No.2	Dingyou-No.8	Baiyan-No.8
Asparagine	71.3	82.6	78	123.3	83.5	59.9
Threonine	40.5	33.9	32.5	52.9	34.2	25.0
Serine	50.4	47.4	44.9	74.8	46.5	34.8
Glutamine	244.7	253.7	246.5	392.7	261.0	192.8
Glycine	18.4	50.0	48.7	80.2	50.3	37.2
Alanine	31.6	48.9	46.1	76.5	48.7	35.6
Cystine	16.5	39.2	35.9	58.6	36.3	27.6
Valine	60.3	56.8	55.6	88.2	57.3	42.9
Methionine	27.7	14.7	15.9	22.0	16.7	12.9
Isoleucine	50.3	38.9	39	61.0	40.8	29.8
Leucine	96.1	77.6	76.4	124.1	80.0	60.4
Tyrosine	52.7	39.7	43.1	69.3	41.3	36.7
Lysine	77.9	59.5	57.7	96.3	58.3	45.8
Histidine	26.2	41.4	38.9	64.3	40.9	30.3
Arginine	33.5	30.3	29.7	47.3	30.5	22.8
Phenylalanine	48.7	62.6	66.3	104.8	68.6	51.2
Proline	104.1	48.4	48.9	85.0	52.2	38.6
Lysine/arginin	2.33	0.66	0.59	0.61	0.60	0.59
Methionin/glycine	1.51	0.29	0.33	0.27	0.33	0.35

**Table 4:** **Fatty acid composition of experimental oats (%)**

	C16:0	C18:0	C18:1	C18:2	C18:3	Unsaturated fatty acids
Jinyan-No.13	18.0	1.1	29.1	47.3	2.0	78.4
Bayou-No.8	19.3	1.6	34.3	40.5	1.5	76.3
Baiyan-No.2	18.6	1.4	31.2	43.5	1.5	76.2
Dingyou-No.8	15.5	1.6	35.8	43.8	1.4	81.0
Baiyan-No.8	16.6	1.8	39.2	37.7	1.5	78.4
Control	8.5	3.4	26.5	52.8	6.7	86.0

Unsaturated fatty acids were the sum of C18:1, C18:2 and C18:3.

**Table 5:** **Tochopherol contents in different oat groups and soybean oil (mg/kg oil)**

	α-Tocopherol	β-Tocopherol	γ-Tocopherol	δ-Tocopherol	^1^Total vitamin E
Jinyan-No.13	88.3	76.5	203.9	108.8	442.3
Bayou-No.8	90.8	-	258.3	128.3	463.5
Baiyan-No.2	83.4	60.0	194.5	136.1	447.2
Dingyou-No.8	114.7	-	197.4	124.9	471.9
Baiyan-No.8	82.6	93.5	196.8	136.0	450.2
Control	28.9	365.6	14.8	19.7	407.3

^1^The data of Total Vitamin E was the sum of α-Tocopherol, β-Tocopherol, γ-Tocopherol and δ-Tocopherol.

- unidentified.

**Table 6:** **Major sterol contents in different oat groups and soybean oil (mg/ kg oil)**

	Campesterol	Stigmasterol	β-Sitosterol	^1^Total sterols
Jinyan-No.13	243.3	183.7	337.6	764.6
Bayou-No.8	275.8	180.7	313.6	770.1
Baiyan-No.2	398.1	85.9	297.4	781.3
Dingyou-No.8	330.3	52.2	377.7	760.2
Baiyan-No.8	344.7	88.2	355.9	788.8
Control	84.2	276.3	322.2	682.7

^1^The data of total sterols was the sum of campesterol, stigmastrol, and β-sitosterol.

The initial body weight, final body weight, body weight gain and food intake for all groups were shown in Table 7. There were no significant differences in any parameters among six groups fed experimental diets for 30 days.

**Table 7:** **Growth parameters and lipid indexes in serum, liver and feces of rats fed the experimental diets for 30 days**

	Control	Jinyan-No.13	Bayou-No.8	Baiyan-No.2	Dingyou-No.8	Baiyan-No.8
Growth Parameters						
Initial BW (g)	270.3 ± 4.1	267.4 ± 6.4	268.7 ± 4.1	282.0 ± 6.7	277.0 ± 7.3	263.4 ± 5.9
Final BW (g)	353.3 ± 11.4	341.9 ± 4.6	338.1 ± 9.2	355.7 ± 8.0	353.6 ± 9.7	357.0 ± 6.3
BW gain (g)	134.0 ± 8.8	129.1 ± 4.0	128.9 ± 6.7	132.3 ± 3.1	129.0 ± 4.7	143.6 ± 7.5
Food intake (g/day)	22.6 ± 0.5	22.2 ± 0.5	22.5 ± 0.3	22.2 ± 0.2	22.3 ± 0.5	21.4 ± 0.3
Serum (mmol/L)						
TC	2.12 ± 0.13^a^	1.88 ± 0.10^b^	1.71 ± 0.12^c^	1.56 ± 0.09^cd^	1.54 ± 0.06^cd^	1.47 ± 0.09^d^
TG	0.71 ± 0.16	0.72 ± 0.17	0.80 ± 0.14	0.58 ± 0.10	0.54 ± 0.07	0.55 ± 0.08
HDL-C	1.35 ± 0.07	1.32 ± 0.06	1.31 ± 0.06	1.35 ± 0.08	1.29 ± 0.05	1.24 ± 0.05
LDL-C	0.46 ± 0.05^a^	0.46 ± 0.04^a^	0.36 ± 0.05^ab^	0.34 ± 0.03^b^	0.35 ± 0.02^b^	0.32 ± 0.02^b^
Liver (μmol/g)						
TC	81.03 ± 2.64^a^	63.04 ± 1.27^c^	63.01 ± 1.56^c^	72.27 ± 2.38^b^	51.77 ± 2.46^d^	47.72 ± 1.76^d^
CE	75.04 ± 2.67^a^	61.22 ± 1.20^b^	61.21 ± 1.54^b^	62.35 ± 3.02^b^	50.23 ± 2.56^c^	44.94 ± 1.45^c^
FC	5.99 ± 0.66^b^	1.82 ± 0.34^c^	1.80 ± 0.35^c^	9.92 ± 1.83^a^	1.54 ± 0.41^c^	2.78 ± 0.52^c^
TG	93.74 ± 7.49	85.38 ± 8.54	100.36 ± 9.23	103.41 ± 7.57	105.81 ± 14.39	107.24 ± 8.53
Feces						
Feces weight (g/d)	1.96 ± 0.03^a^	2.13 ± 0.03^a^	2.07 ± 0.03^a^	2.07 ± 0.04^a^	2.40 ± 0.05^b^	2.04 ± 0.04^a^
Total fat (g/d)	0.148 ± 0.007	0.150 ± 0.006	0.159 ± 0.005	0.148 ± 0.005	0.152 ± 0.006	0.136 ± 0.005
TC (μmol/d)	66.3 ± 5.9^a^	49.3 ± 3.4^b^	54.6 ± 5.1^b^	52.7 ± 3.6^b^	45.8 ± 3.6^c^	43.4 ± 3.5^c^
Bile acid (μmol/d)	47.5 ± 2.14^a^	54.7 ± 2.31^b^	50.5 ± 2.01^a^	56.1 ± 2.41^b^	61.3 ± 4.10^b^	53.7 ± 1.98^b^

Data are means ± SE (*n* = 7). Values not sharing a common letter are significantly different at *P* < 0.05, by Tukey–Kramer multiple comparisons. *BW,* body weight; *TG,* triacylglycerol; *CHOL,* cholesterol; *HDL-C,* HDL-cholesterol; *LDL-C,* LDL-cholesterol.

Table 7 also showed the serum, liver and fecal lipids profiles of rats after treating with experimental diets for 30 days. Dietary Baiyan-No.2, Dingyou-No.8, and Baiyan-No.8 significantly reduced both serum TC and LDL-C levels compared with control group, while dietary Jinyan-No.13 and Bayou-No.8 only decreased serum TC levels significantly. Serum HDL-C and TG concentrations of five oat groups were not significantly different from that of control group. The concentrations of liver TC and CE in oat groups were significantly reduced compared with control group. Besides Baiyan-No.2 group, the contents of liver FC were also significantly decreased in other four oat groups. Liver TG contents and fecal lipids excretion were not changed. In addition, fecal bile acid excretion was increased in oat groups, while the fecal cholesterol excretion was decreased. Fecal weight was increased in Dingyou-No.8 group, but not in other four oat groups.

**Figure 2:**
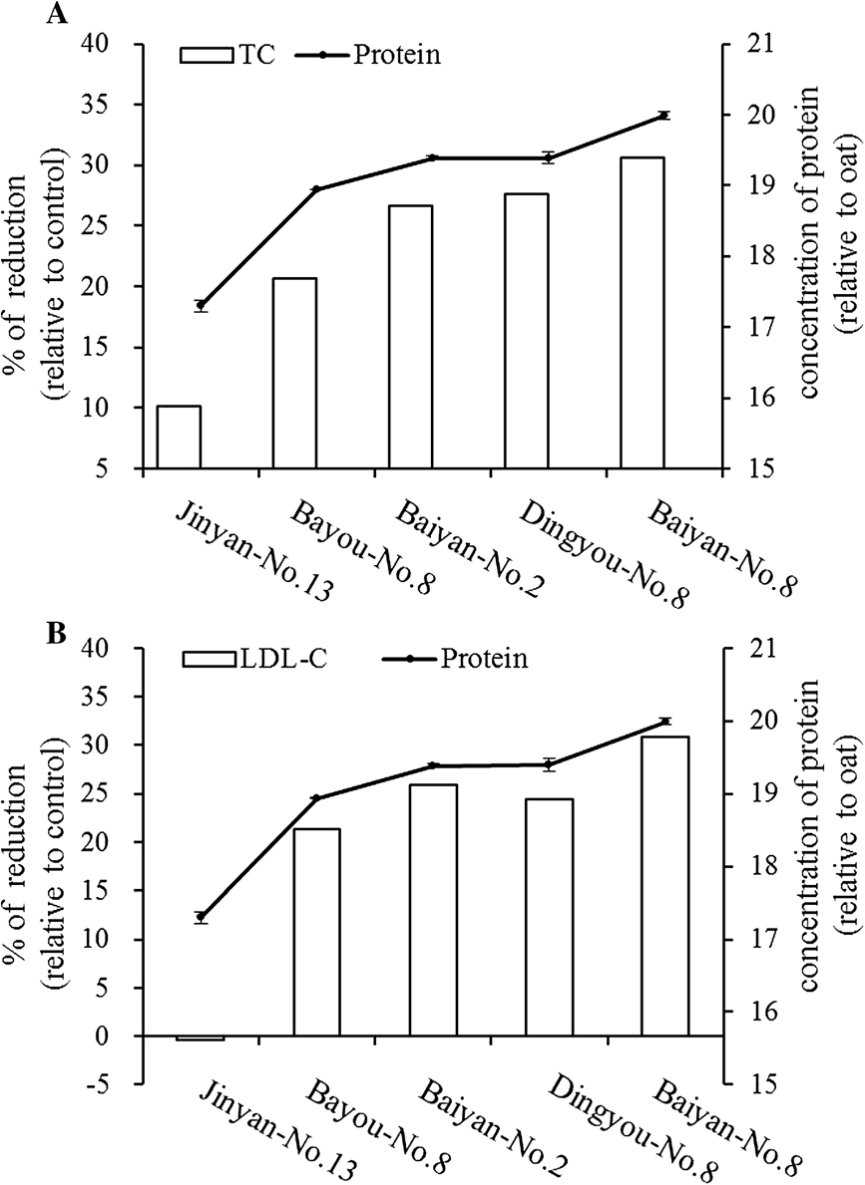
**Comparison between the contents of oat proteins and the percentage reduction of serum TC (A) and LDL-C (B) in rats fed experiment diets.** The contents of oat proteins are expressed as mean ± SD (*n* = 3). *TC,* total cholesterol; *LDL-C,* LDL-cholesterol.

**Figure 3:**
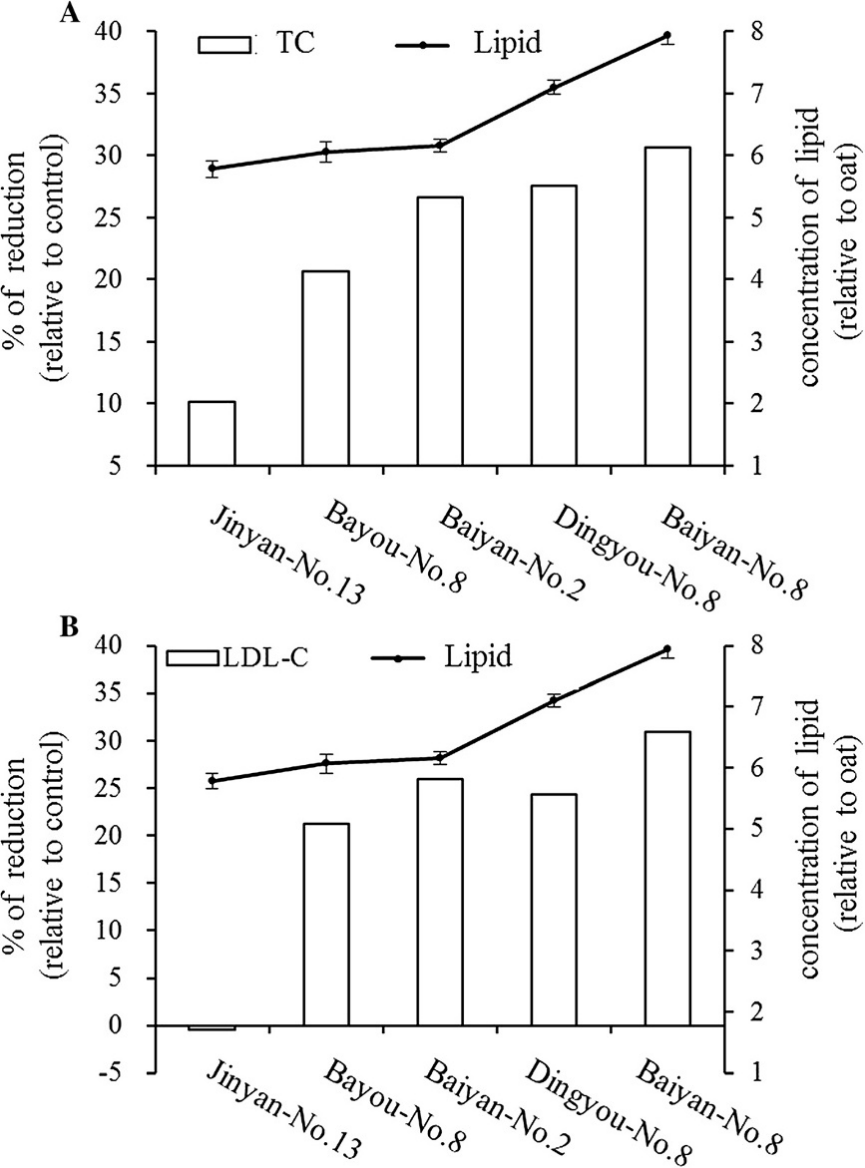
**Comparison between the contents of oat lipids and the percentage reduction of serum TC (A) and LDL-C (B) in rats fed experiment diets.** The contents of oat lipids are expressed as mean ± SD (n = 3). *TC,* total cholesterol; *LDL-C,* LDL-cholesterol.

Comparison between oat proteins/oat lipids and the percentage reduction of serum total cholesterol and LDL-cholesterol were shown in Figure 2 and Figure 3. The percentage reduction of total cholesterol and LDL-cholesterol were higher after rats obtained higher contents of oat proteins. The similar tendency was also found between oat lipids and the percentage reduction of serum total cholesterol and LDL-cholesterol. It was indicated that the percentage reduction of serum total cholesterol and LDL-cholesterol depended on the contents of proteins and lipids, and the impact from high to low in order was: Baiyan-No.8, Dingyou-No.8, Baiyan-No.2, Bayou-No.8, and Jinyan-No.13.

## Discussion

It has been demonstrated that intake of oat products or oat bran could decrease cholesterol levels of serum in human and animals [21, 22]. In the present study, we chose five oat varieties with different contents of proteins and lipids, but similar content of β-glucan, to investigate the cholesterol-lowing effects of oat components in rats. As the results, all tested oat varieties played the cholesterol-lowering effects, but the hypocholesterolemic degree among different oat groups was significantly different (Table 7). This result indicated that other components in oat were responsible for the observed effects. Moreover, the ability of different oat varieties to reduce serum total cholesterol and LDL-cholesterol concentrations increased with the increase of the proteins and lipids contents, but had nothing to do with starch contents (Figure 2 and Figure 3). These findings suggest that the proteins and lipids in oats may play the cholesterol-lowering effects.

In fact, numerous studies have demonstrated the cholesterol-lowering effect of plant proteins in soybean [12], rice [14, 15] and buckwheat [23, 24] in animals fed cholesterol enriched diet. Moreover, Rajamohan et al. discovered that sesame protein showed stronger reduction of serum cholesterol level than casein, probably due to its low ratio of lysine/arginine (0.67) [25]. Similarly, Morita et al. found that cholesterol-lowering effects of soybean protein depended on its low ratio of methionine/glycine (0.323) into rats fed a cholesterol-free purified diet [12]. In the present study, the ratio of lysine/arginine and methionine/glycine of oats proteins were lower than that of casein, but similar to sesame proteins and soybean proteins (Table 3). These findings could account for the cholesterol-lowering effect of oat was closely related to its protein contents, namely, the higher contents of oat proteins, the lower total cholesterol and LDL-cholesterol levels of serum were observed in rats fed a hypocholesterolemic diet.

Rice bran oil and its consumption have been reported to decrease cholesterol levels of serum significantly in human and animal [26, 27]. Richard et al. concluded that oat bran affected cholesterol metabolism through lipids and non-starch polysaccharides in adult male rats fed a cholesterol-free synthetic diet [28]. The present study showed that the higher contents of oat lipids led to the lower total cholesterol and LDL-cholesterol levels of serum in rats. Besides oat proteins, lipids were also involved in the cholesterol-lowering effect of oat. Furthermore, it was reported that dietary monounsaturated fatty acid and polyunsaturated fatty acid diets resulted in lower serum total cholesterol and low-density-lipoprotein cholesterol in human [29]. Our study found that the rats fed hypercholesterolemic diets supplemented with oats ingested less amount of unsaturated fatty acids than control group (Table 4), suggesting that the hypolipidemic effect of oat lipids was not simply dependent on its fatty acid composition.

Previous studies reported that supplementation with vitamin E could reduce plasma levels of LDL and oxidized LDL [30], meanwhile inhibit oxidation of LDL cholesterol and prevent atherosclerosis [31]. The most abundant phytosterol of oat were campesterol, stigmastrol, and β-sitosterol [32]. Numerous studies reported that plant sterols had cholesterol-lowering effect by inhibiting the absorption of intestinal cholesterol [33]. Plant sterols could displace cholesterol from bile salt micelles and restrict the micellar solubility of cholesterol, which accounted for the inhibitory effect on cholesterol absorption [33–35]. Our results showed that the concentrations of total vitamin E and total plant sterols among five oat groups were similar, but they were higher than that in the control group (Table 5 and Table 6). Together with the really low contents of vitamin E and plant sterols in the hypercholesterolemic diets in the present study, it was impossible to illustrate which of these two components was responsible for the hypolipidemic effects alone.

Numerous studies demonstrated that dietary red palm oil supplementation offered significant protection against ischemia/reperfusion-induced injury in the isolated perfused rat heart by improving functional recovery [36–38]. The oleic, linoleic, tocopherol and tocotrienol, sterols, squalene, and CoQ10 were considered to be the main functional components [36, 39]. It has been reported that pretreatment with a combination of vitamins A and E showed protection against venous ischemia/reperfusion-induced injury, but these vitamins were not effective when they were used as single agents [40]. Therefore, we speculate that the hypocholesterolemic properties of oat lipids could not be attributed to one single component, but to the combination of oleic acid, vitamin E, or plant sterols.

Inhibition of lipids absorption in small intestines and increase of excretion of fecal bile acids in the liver were thought to be the hypocholesterolemic mechanisms of oat [41, 42]. In this study, dietary oats resulted in the significant decrease of TC and CE concentrations in liver, and oat groups increased daily excretion of bile acids, but did not promote the excretion of fecal cholesterol. It indicated that diet oats decreased serum and liver cholesterol concentrations by promoting the excretion of fecal bile acids.

It has been reported that intake of fish oil and fish proteins showed different ways to regulate cholesterol levels in serum and liver. Dietary fish proteins decreased serum and liver cholesterol through increasing fecal cholesterol, bile acids excretion and liver cholesterol 7α-hydroxylase expression level, while dietary fish oil decreased liver cholesterol perhaps due to the suppression of cholesterol synthesis through a decrease in the 3-hydroxy-3methylglutaryl-coenzyme A reductase expression level . In our study, the concentrations of liver cholesterols and/or fecal bile acids were not affected by proteins and lipids, which may be due to different ways of β-glucan, proteins and lipids to regulate cholesterol levels. However, the exact mechanisms of cholesterol-lowering effects of β-glucan, proteins and lipids in oat still need further researches.

## Conclusions

The present study showed that dietary oats decreased the concentrations of plasma and liver cholesterol in Wistar-Lewis rats fed with a hypercholesterolemic diet by increasing daily excretions of fecal bile acids. Dietary oats improved hypercholesterolemia, not only attribute to β-glucan but also related to the proteins and lipids. Furthermore, the cholesterol-lowering effect of proteins in oats could be attributed to its more reasonable lysine/arginin and methionin/glycine ratio and the hypocholesterolemic properties of oat lipids due to the combination of oleic acid, linoleic, vitamin E, or plant sterols.
